# Determinants of time to decannulation and predictors of early weaning from tracheostomy: a multicenter, retrospective Italian cohort study

**DOI:** 10.1016/j.aicoj.2026.100050

**Published:** 2026-03-17

**Authors:** Dejan Radovanovic, Fabiano Di Marco, Michele Mondoni, Claudia Crimi, Andrea Gramegna, Marina Gatti, Juan Camilo Signorello, Federico Raimondi, Cristina Albrici, Giorgio Morana, Francesco Bruno Arturo Blasi, Pierachille Santus

**Affiliations:** a Department of Biomedical and Clinical Sciences, Università Degli Studi di Milano, Italy; b Division of Respiratory Diseases, Ospedale L. Sacco, ASST Fatebenefratelli-Sacco, Milano, Italy; c Coordinated Research Center on Respiratory Failure, Università degli Studi di Milano, Milano, Italy; d Department of Health Sciences, Università Degli Studi Di Milano, Milan, Italy; e Respiratory Unit, ASST Papa Giovanni XXIII Hospital, Bergamo, Italy; f Respiratory Unit, Department of Health Sciences, ASST Santi Paolo e Carlo, Università Degli Studi di Milano, Milano, Italy; g Department of Clinical and Experimental Medicine, University of Catania, Catania, Italy; h Respiratory Intensive Care Unit, Respiratory Medicine Unit, Policlinico "G. Rodolico-San Marco" University Hospital, Catania, Italy; i Department of Pathophysiology and Transplantation, Università Degli Studi di Milano, Italy; j Respiratory and Cystic Fibrosis Unit, Fondazione IRCCS Ca’ Granda Ospedale Maggiore Policlinico Milano, Milano, Italy

**Keywords:** Decannulation, Weaning, Tracheostomy, Tube capping, Bronchoscopy

## Abstract

**Background:**

Weaning from tracheostomy is largely left to experts’ opinion. Shared and validated protocols for decannulation are lacking, and procedures during the weaning process depend upon clinical judgement. Determinants of tracheostomy decannulation in real life are largely unknown.

**Methods:**

This was a retrospective, observational, multicenter study. Patients that underwent endotracheal intubation, percutaneous or surgical tracheostomy and at least a decannulation trial between 2017 and 2023 were recruited from five academic hospitals in Italy. Clinical characteristics, procedures, pre-decannulation respiratory and biochemistry parameters, respiratory support, Quantitative semi-Quantitative clinical score (QsQ), and in-hospital outcomes were collected. Patients were operationally divided in early (<20), average (20–40), and late (>40 days) decannulation. The aim was to assess predictors of faster decannulation. Secondary outcomes included: weaning failure, instrumental procedures during weaning, accuracy of QsQ criteria for decannulation failure.

**Results:**

The final analysis included 191 patients (26.7% males, median age 63 years), of which 79.6% had at least one comorbidity and 61.2% were intubated for Coronavirus Disease 2019 pneumonia. Decannulation was successful in 183 patients (95.8%) and failed in 8 (4.2%). Early, average and late decannulation was observed in 23.3, 31.7 and 45% of patients, respectively. Weaning was faster if patients underwent bronchoscopy (Log-rank P = 0.044), and longer if swallowing efficiency was assessed (Log-rank P = 0.001). Reduction of cannula caliber (OR 4.224, 95%CI: 1.037–17.207; P = 0.044) predicted earlier decannulation, while swallowing assessment predicted slower decannulation (OR 0.161, 95%CI: 0.037−0.694; P = 0.014). Patients’ baseline clinical characteristics and instrumental procedures didn’t differ in patients that failed and that succeeded weaning. Successes had a lower bronchial secretion burden (P = 0.012) and tended to develop less frequently tracheal stenosis (P = 0.072). Sufficient data availability for QsQ score calculation were limited to some of the major and minor criteria.

**Conclusions:**

Specific procedures were associated with reduced time to decannulation, however weaning rarely failed likely because of late weaning initiation. Results generalizability could be limited by younger age, low comorbidity burden and high prevalence of COVID-19 pneumonia. Trials investigating per-protocol weaning to detect the most performant screening procedures are required.

**Trial registration:**

The study protocol has been registered and approved by ClinicalTrials.gov February the 28th 2022 (NCT05271786).

## Introduction

Patients requiring prolonged mechanical ventilation and difficult weaning represent a significant healthcare burden, accounting for more than 13% of ventilated patients and 37% of total Intensive Care Unit (ICU) costs [1,2]. Tracheostomy is one of the procedures most frequently performed in patients requiring prolonged mechanical ventilation [3]. Tracheostomy indications, timing, techniques, and short to long term benefits have been partly explored, but still debated [[4], [5], [6], [7], [8]]. Indeed, one of the major clinical and research gaps consists of weaning from tracheostomy, that is largely left to unvalidated protocols, experts’ opinion, and clinical judgement [[8], [9], [10], [11]]. Decannulation refers to the permanent removal of the tracheostomy cannula, and usually encompasses several steps (weaning), involving a multidisciplinary team that includes pulmonologists, critical care physicians, phoniatricians, speech therapists, and respiratory physiotherapists. A timely decannulation favors patient recovery, reduces length of stay, and lowers the risk of potentially life-threatening complications, such as lower respiratory tract infections, aspiration pneumonia, bleeding, tracheal stenosis and tracheomalacia, swallowing and speech impairment [8]. So far, investigations about changes of upper airway physiology and mechanics while the tracheostomy tube is in place and after decannulation are still conflicting [12]. Accordingly, several decannulation protocols have been proposed, but controversies still exist in terms of when and how perform bronchoscopy, swallow testing, time to reduce the cannula caliber, or which physiological parameters should be considered to undertake tube capping or decannulation [11,[13], [14], [15]]. Recently, Santus et al. proposed the first predictive decannulation score (the Quantitative semi-Quantitative clinical score, QsQ) [8]. The score however, to date, was never validated in clinical practice.

The aim of the present study was to describe the instrumental procedures performed during the weaning process, investigate predictors of faster decannulation, weaning failure, and to test the clinical feasibility and accuracy of the QsQ score in real life clinical settings.

## Methods

This was a retrospective, multicenter, cohort study. Consecutive adult patients that underwent tracheostomy from January 2017 until April 2023 were recruited from four academic Italian weaning centers and intermediate respiratory care units: L. Sacco University Hospital (Milan, Italy), ASST Papa Giovanni XXIII Hospital (Bergamo, Italy), San Paolo Hospital – ASST Santi Paolo e Carlo (Milan, Italy), Policlinico "G. Rodolico-San Marco" University Hospital (Catania, Italy) and the Respiratory and Cystic Fibrosis Unit of Fondazione IRCCS Ca’ Granda Ospedale Maggiore Policlinico (Milan, Italy).

Inclusion criteria were: (i) age ≥18 years old; (ii) percutaneous or surgical tracheostomy performed for repeated weaning failure or difficult weaning from invasive mechanical ventilation (IMV), prolonged IMV (≥21 days), inability to manage airway secretions, low level of consciousness (e.g. prolonged sedation); (iii) at least one decannulation trial during the hospital stay. Patients were excluded if underwent a permanent tracheostomy, elective tracheostomy for neck masses, malignancy, or upper airway obstruction, and/or have been tracheostomized more than once. Patients with a confirmed history of degenerating neuromuscular disease (NMD) prior to ICU admission such as amyotrophic lateral sclerosis, or patients admitted for or with a positive history of post-traumatic injury or acute brain injury were also excluded. Patients with acute brain injury were considered suffering from any of the following: stroke (ischemic stroke, intracranial and subarachnoid hemorrhage), traumatic acute brain injury, hypoxemic-ischemic encephalopathy following cardiac arrest. Acute NMDs such as Guillain–Barrè syndrome or myasthenia gravis diagnosed during the ICU stay or leading to endotracheal intubation did not represent exclusion criteria. Patients with ICU-acquired weakness were not excluded from the study.

### Study objectives

The primary objective of the study was to assess determinants of the duration of weaning process and identify predictors of early decannulation.

Secondary objectives included: (i) the description of the procedures conducted during the weaning process, (ii) the proportion and characteristics of patients that failed decannulation, (iii) accuracy and feasibility of the QsQ score [8].

### Data collection

Patients’ medical records were reviewed to extrapolate the following variables: anthropometrics, comorbidities, reason for endo-tracheal intubation (ETI), diagnostic procedures including bronchoscopy, swallow testing and cough efficiency assessment, need and daily frequency of bronchial aspirations, presence of tracheal stenosis, type of respiratory support before and after decannulation, and procedures related to the weaning process such as characteristics of tracheostomy cannula (inner diameter, presence of cuff or fenestration), tube capping and reduction of cannula caliber. Gas exchange parameters, vital signs and level of consciousness in the last 24 h before decannulation were collected. Date of hospitalization, ETI and tracheostomy decannulation were registered. ICU-acquired weakness could represent a potential confounding factor able to influence timing of decannulation, but due to the heterogeneity of diagnosis, the unavailability of disease severity and degree of respiratory muscular involvement, it was not included among the collected variables. The QsQ clinical score was calculated as previously suggested [8] (Supplementary Table S1). Details on QsQ criteria and score threshold are reported in the supplementary file.

### Study definitions

A decannulation trial was defined as the procedure to remove the tracheostomy cannula and sealing the stoma with a dressing or a tracheostomy retainer, independently of the presence of tube capping, type of respiratory support before and following removal. Accidental dislodgement of the tracheostomy tube was accepted as long as the conditions pre-displacement were restored. Self-decannulated patients and patients experiencing accidental decannulation followed by no re-incannulation were excluded from the study. Decannulation failure was defined as the need to re-position a tracheostomy cannula for any reason or need for ETI in the 48 h following decannulation [16]. A tube capping attempt (the positioning of a cap that mechanically impedes the respiration through the tracheostomy cannula and forces airflow through the upper airways) was considered if capping was tolerated >12 h straight. A tube capping trial success was considered when tube capping was tolerated ≥24 h straight as suggested by Stelfox et al. [16] and was considered for QsQ criteria. Any type of cannula caliber reduction was considered, including when the cuffed tracheostomy cannula was replaced with a non-cuffed (fenestrated or non-fenestrated) cannula. Tracheal stenosis was assessed as reported by Perroni et al. [17]. Swallowing assessment was considered part of the weaning procedures when performed by a phoniatrician/otolaryngologist by means of a laryngoscope. Time to decannulation was the time between tracheostomy and decannulation; hospital stay was the time the patients spent in the hospital (including both ICU and respiratory ward) from day of hospitalization to hospital discharge; duration of IMV was the time in days the patient spent receiving invasive mechanical ventilation while intubated (the time to conclude weaning from IMV while the patient was tracheostomized was not considered for this period); time from decannulation to discharge was the time period the patient spent in hospital from the day of decannulation until hospital discharge. The ratio between time spent in the hospital between decannulation and hospital discharge and duration of the decannulation process (i.e. time to decannulation) was used to describe the influence of decannulation speed and possibility hospital discharge (day of hospital discharge – day of decannulation / day of decannulation – day of tracheostomy).

### Statistics

A sample size calculation, due to the explorative nature of the study, was not attainable. However, one of the previous largest observational studies targeted on decannulation have involved 126 patients [15]. The size of our cohort has been targeted to reach at least 189 patients, therefore increasing previous cohorts by 50%. Qualitative and quantitative variables were described as frequencies, mean (standard deviation – SD) or median (inter quartile range – IQR) depending on their parametric distribution. Normality of distribution was assessed with the Kolmogorov-Smirnov test. Chi-squared and Fisher exact test were used to compare categorical variables; continuous variables were compared with the Student t-test, Mann–Whitney U or Kruskal-Wallis tests depending on their distribution, as appropriate. Patients were operatively divided by time to decannulation: “early”, “average” and “late” decannulation group, that were compared by clinical characteristics, weaning procedures and outcomes. Considering the observed overall duration of the weaning process, three operative timings were identified: <20 days (early group), 20–40 days (average group) and >40 days (late group). Logistic regression analysis preceded by the assessment of collinearity was performed to assess the risk to be part of the “early group”. Considering the limited number of decannulation failures, predictors for failure of decannulation were not computed. Kaplan–Meier survival curves with Wilcoxon–Breslow–Gehan test were assessed to test the time to decannulation in patients that underwent bronchoscopy, cannula caliber change, swallowing assessment and were aged > or ≤65 years old. A two-tailed p-value <0.05 was considered statistically significant. Missing data were not imputed. Statistical analyses were performed with IBM SPSS, Statistics for Windows version 21.0 (Armonk, NY; IBM Corp).

## Results

A total of 196 patients were enrolled and 191 patients (26.7% males, median [IQR] age 63 [54−70] years) were included in the final analysis (Table 1). Eight patients (4.2%) failed decannulation, while 183 patients (95.8%) succeeded. Decannulation failure occurred due to respiratory distress (n = 4), obstructive tracheal granuloma (n = 3) and vocal cord paralysis (n = 1). Reason for patients’ exclusion are detailed in the supplementary material.

**Table 1:** Anthropometrical and clinical characteristics of the study cohort.

	All	Success	Failure	
Variable	N = 191	N = 183	N = 8	*p*-value
Males, n (%)	51 (26.7)	49 (27)	2 (25)	0.637
Age (years), – median (IQR)	63 (54−70)	63 (54−70)	64 (35−72)	0.717
IMV duration (days), median (IQR)	15 (8−19)	15 (8−19)	16 (9−16)	0.751
Comorbidities				
≥1 comorbidity, n (%)	152 (79.6)	146 (79.8)	6 (75) (	0.718
Obesity, n (%)	32 (16.8)	30 (16.4)	2 (25)	0.623
Diabetes, n (%)	41 (21.5)	39 (21.3)	2 (25)	0.682
Diabetes type II, n (%)	31 (16.2)	30 (16.4)	1 (12.5)	0.629
Diabetes type I, n (%)	10 (5.2)	9 (4.9)	1 (12.5)	
Ischemic heart disease, n (%)	29 (15.2)	28 (15.3)	1 (12.5)	0.651
COPD, n (%)	25 (13.1)	23 (12.6)	2 (25)	0.281
Chronic heart failure	18 (9.4)	17 (9.2)	1 (12.5)	0.546
HFrEF	11 (5.7)	10 (5.5)	1 (12.5)	0.468
HFpEF	7 (3.6)	7 (3.7)	0	–
Solid tumors, n (%)	16 (8.4)	15 (8.2)	1 (12.5)	0.510
Neurological disease, n (%)	14 (7.3)	14 (7.7)	0	–
Rheumatological disorder, n (%)	10 (5.2)	10 (5.5)	0	–
QsQ score, median (IQR)	50 (45−55)	50 (45−55)	50 (36−55)	0.860
Reason for tracheostomy*				
Need for prolonged IMV, n (%)	53 (27.9)	53 (29.1)	0	0.439
Weaning failure, n (%)	131 (68.9)	123 (67.6)	8 (100)	
Other, n (%)	6 (3.2)	5 (3.2)	0	
Tracheostomy technique**				
Griggs, n (%)	31 (17.9)	30 (18.1)	1 (14.3)	
Percutwist, n (%)	3 (1.7)	3 (1.8)	0	
Ciaglia-Blue Rhino, n (%)	86 (49.7)	81 (48.8)	5 (71.4)	0.956
Surgical, n (%)	24 (13.9)	23 (13.8)	1 (14.2)	
Percutaneous not specified, n (%)	29 (16.7)	28 (16.9)	1 (14.2)	
Mucus/secretions***				
Absent, n (%)	40 (21.2)	39 (21.3)	1 (12.5)	**0.012**
Managed by patient, n (%)	114 (60.3)	112 (61.2)	2 (25)	
>1 aspirations/day, n (%)	23 (12.2)	20 (10.9)	3 (37.5)	
>3 aspirations/day, n (%)	12 (6.3)	10 (5.5)	2 (25)	
Respiratory support before decannulation†				
Room air, n (%)	60 (31.9)	54 (29.5)	6 (75)	**0.014**
Nasal cannula O2, n (%)	81 (43.1)	80 (43.7)	1 (12.5)	0.141
Tracheolife O2, n (%)	4 (2.1)	4 (2.2)	0	1.000
HFNC, n (%)	29 (15.4)	28 (15.3)	1 (12.5)	1.000
Venturi mask, n (%)	6 (3.2)	6 (3.3)	0	1.000
Mechanical Ventilation, n (%)	8 (4.2)	8 (4.4)	0	1.000

Comorbidities are listed based on prevalence. Only comorbidities with >5% prevalence are shown. Weaning failure included either the need for re-intubation after an extubation or (repeated) failure of a spontaneous breathing trial. A complete list of comorbidities is reported in Supplementary Table S1. Data are reported as frequencies (prevalence) and medians (inter quartile range).

COPD = Chronic Obstructive Pulmonary Disease; HFrEF = heart failure with reduced ejection fraction; HFpEF = heart failure with preserved ejection fraction; HFNC = High Flow Nasal Cannula; IMV = invasive mechanical ventilation; QsQ = Quantitative semi quantitative score.

* Information was available for n = 190.

** Information was available for n = 173.

*** Information was available for n = 189.

† Information was available for n = 188.

### Patients’ characteristics, procedures and clinical outcomes

Clinical characteristics of the study cohort are reported in Table 1 and Supplementary Table S2. Severe community acquired pneumonia (74.3%) and shock (12.6%) were the most frequent reasons for instituting mechanical ventilation; COVID-19 pneumonia accounted for 61.2% of cases (Supplementary Table S3). Weaning failure from mechanical ventilation (68.9%) was the most common reason for tracheostomy (Table 1). Patients that succeeded and failed did not differ in terms of anthropometrics, comorbidities, reason for ETI, reason for tracheostomy, tracheostomy techniques, characteristics and calibers of tracheostomy cannulas and weaning procedures (Table 1, Supplementary Tables S2–S4). Failure group had a higher proportion of patients that presented accessory muscle activation before decannulation (P = 0.015). Three patients that failed (37.5%) had a sternomastoid activation before decannulation, compared with three patients that succeeded (1.7%; P < 0.001). Pre-decannulation gas exchange and vital signs did not differ between groups (Supplementary Table S5). During the hospital stay, patients that failed decannulation needed to be aspirated more frequently compared with patients that succeeded (P = 0.012; Table 1). Bronchoscopy, swallowing and cough efficiency were performed in 76%, 67% and 92.1% of patients. Tube capping was tested in 83.8% of the study cohort.

The median (IQR) QsQ score was 50 (45−55) points and did not differ between patients that succeeded and patients that failed (50 (45−55) vs 50 (36−55); P = 0.860). Four patients died during the hospital stay, 3 in the success group, and 1 in the failure group (P = 0.159, Supplementary Table S4). Causes of death were: severe hospital acquired pneumonia causing terminal type 2 respiratory failure(n = 2), sepsis due to candida infection (n = 1), malignant arrhythmia leading to cardiac arrest (n = 1).

### Time to decannulation

**Fig. 1:**
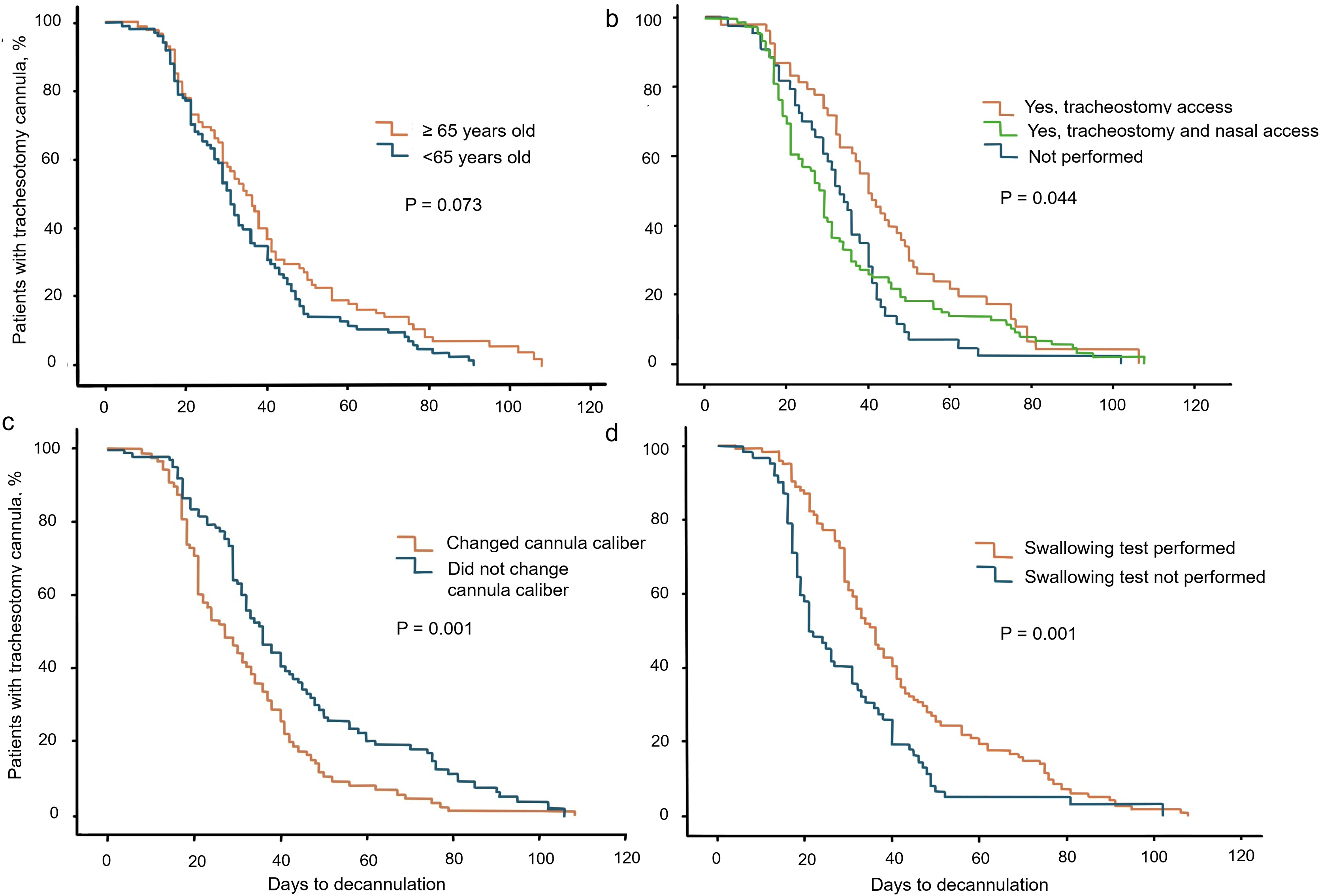
Time to decannulation depending on weaning procedures. Kaplan–Meier curves showing time to decannulation in the study cohort divided by age (panel a), bronchoscopy execution (panel b), change of cannula size (panel c) and swallowing efficiency testing (panel d). P-values are log-rank test.

Of the 183 patients that succeeded weaning, decannulation date was available in 180 cases. The median (IQR) time to decannulation was 31.5 (21–46) days. Forty-two (23.3%) patients were decannulated in <20 days (early group), 81 (45.0%) patients between 20 and 40 days (average group), and 57 (31.7%) patients were weaned in >40 days (late group) (Table 2). Clinical characteristics and comorbidities largely overlapped between groups. Obesity was less prevalent in the early group (P = 0.026). In terms of reasons for ETI, cardiogenic shock was more frequently observed in the late group (P = 0.027; Table 2). Performing bronchoscopy through nasal access and cannula caliber reduction reduced time to decannulation (Fig. 1b-1c and Table 3). Frequent bronchial aspirations and the need for swallowing assessment significantly increased weaning length (P = 0.048, Table 3 and Fig. 1d). Swallowing assessment was significantly more frequent in patients that did not perform nasal bronchoscopy compared with patients that performed nasal bronchoscopy (79.8% vs 53.3%; P < 0.001). Signs of accessory respiratory muscles activation pre-decannulation did not influence time to decannulation (Table 3), while PaO_2_ pre-decannulation was significantly higher in patients that were decannulated earlier compared with other groups (P = 0.011; Table 3), although the observed differences were not clinically relevant. Hospital stay was significantly shorter and time from decannulation to discharge tended to be reduced in the early decannulation group (Table 3). Moreover, the proportion of time spent in the hospital between decannulation and hospital discharge in respect to the length of the decannulation process (time to decannulation) was progressively less in early (0.26 (0.14−0.38)) vs average (0.20 (0.13−0.31)) vs late (0.13 (0.06−0.26)) decannulation groups, indicating that decannulation was significantly associated with hospital discharge (P < 0.001).

**Table 2:** Patients’ characteristics based on the duration of weaning from tracheostomy.

Variable	Early n = 42	Average n = 81	Late n = 57	*p-*value
Males	11 (26.2)	22 (27.2)	14 (24.6)	0.943
Age, years	63 (54-73)	62 (53-70)	65 (56-72)	0.472
Comorbidities				
≥ 1 comorbidity	35 (83)	68 (84)	46 (81)	0.615
Arterial hypertension	21 (50)	45 (55.6)	29 (50.9)	0.793
Ischemic cardiac disease	7 (16.7)	8 (9.9)	12 (21.1)	0.183
Chronic heart failure	4 (9.5)	8 (9.9)	6 (10.6)	0.812
HFrEF	2 (4.8)	5 (6.2)	4 (7)	0.520
HFpEF	2 (4.8)	3 (3.7)	1 (1.8)	0.264
Diabetes mellitust type 2	4 (9.5)	12 (14.8)	12 (21.1)	0.428
COPD	5 (11.9)	9 (11.1)	8 (14.0)	0.873
Solid tumors	4 (9.5)	5 (6.2)	5 (8.8)	0.760
SARS-CoV-2	25 (59.5)	54 (66.7)	37 (64.9)	0.450
Obesity	3 (7.1)	20 (24.7)	7 (12.3)	**0.026**
Atrial fibrillation	0	0	4 (7)	–
Cerebrovascular disease	3 (7.1)	1 (1.2)	3 (5.3)	0.233
Diagnosis at ICU admission				
Pneumoccocal CAP	5 (11.9)	7 (8.6)	3 (5.3)	0.493
Influenza CAP	2 (4.8)	4 (4.9)	0	0.237
Other CAP	1 (2.4)	0	0	0.192
Hospital acquired pneumonia	2 (4.8)	3 (3.7)	4 (7.0)	0.677
Aspiration pneumonia	2 (4.8)	0	0	**0.036**
Idiopathic pulmonary fibrosis	0	0	1 (1.8)	0.338
Septic Shock	2 (4.8)	5 (6.2)	4 (7.0)	0.898
Cardiogenic shock	1 (2.4)	2 (2.5)	7 (12.3)	**0.027**
Aortic dissection	0	3 (3.7)	0	0.155
COPD exacerbation	0	2 (2.5)	1 (1.8)	0.597
Asthma exacerbation	1 (2.4)	0	0	–
Tetanus	0	1 (1.2)	0	–
Meningitis	1 (2.4)	1 (1.2)	2 (3.6)	0.669
Pneumothorax	0	1 (1.2)	0	–

The duration of weaning from tracheostomy was available in 180 out of 183 patients that succeeded decannulation. Early: <20 days; average: 20–40 days; late: >40 days. Data are expressed as frequencies (%). Age is expressed as median (IQR). P-values are 2-sided. Bonferroni correction was applied for multiple comparisons for independent sample in case of non-normally distributed data.

CAP = Community acquired pneumonia; COPD = Chronic Obstructive Pulmonary Disease; HFrEF = heart failure with reduced ejection fraction; HFpEF = heart failure with preserved ejection fraction; SARS-CoV-2 = Severe Acute Respiratory Syndrome Coronavirus type 2.

**Table 3:** Weaning procedures, clinical and gas exchange variables pre-decannulation based on the duration of weaning from tracheostomy.

Variable	Early n = 42	Average n = 81	Late n = 57	*p*-value
Procedures and variables during weaning				
Swallowing assessement*	19 (42.2)	58 (70.7)	49 (80.3)	**<0.001**
Swallowing efficacy				
No impairment (n = 81)	14 (73.7)	37 (63.8)	30 (61.2)	0.620
Mild dysphagia (n = 25)	4 (21)	12 (20.7)	9 (18.4)	
Moderate-severe dysphagia (n = 18)	1 (5.2)	7 (12.1)	10 (20.4)	
Caliber reduction	28 (62.2)	39 (47.6)	22 (36.1)	**0.029**
Tube capping	35 (77.8)	70 (86.4)	53 (86.9)	0.408
Efficient cough	42 (93.3)	77 (93.9)	55 (90.2)	0.683
Peak cough flow, L/min**	–	310 (185-310)	255 (129-308)	0.544
Secretions:				
Absent	11 (24.4)	20 (24.4)	6 (11.9)	
Managed by the patient	28 (66.7)	50 (61.0)	33 (55.9)	**0.041**
>1 aspirations/day	2 (4.8)	6 (7.4)	12 (21.1)	
> 3 aspirations/day	1 (2.2)	5 (6.1)	4 (7.0)	
Bronchoscopy:				
Not performed	9 (20.0)	23 (28.1)	12 (19.7)	
Tracheostomy access	7 (15.6)	21 (25.6)	26 (42.6)	**0.015**
Nasal access	29 (64.4)	38 (46.3)	23 (37.7)	
Nasal and tracheostomy access	7 (16.7)	20 (24.7)	23 (40.3)	**0.024**
Tracheal stenosis	2 (4.5)	3 (3.7)	6 (10)	0.266
Tracheal stenosis (severity, %)	30 (30-30)	–	50 (45-75)	0.068
Respiratory support pre-decannulation				
Room air	10 (23.8)	26 (32.1)	16 (28.1)	0.602
Nasal cannula	20 (47.6)	32 (40)	28 (45.9)	0.530
Tracheolife	1 (2.3)	1 (1.3)	2 (3.3)	0.683
High flow nasal cannula	6 (16.7)	15 (18.8)	7 (11.5)	0.556
Venturi Mask	3 (6.8)	1 (1.3)	2 (3.3)	0.221
Mechanical ventilation	1 (2.3)	4 (5)	2 (3.3)	0.766
Gas exchange pre-decannulation***				
pH	7.45 (7.44-7.48)	7.46 (7.43-7.48)	7.45 (7.43-7.48)	0.850
PaO2, mmHg	93 (73-106)	75 (65-91)	81 (72-98)	**0.011**
PaCO2, mmHg	39 (36-41)	39 (36-42)	40 (36-44)	0.921
FiO2, %	27 (21-30)	24 (21-30)	24 (21-24)	0.190
QsQ score	50 (40-53)	50 (45-55)	50 (45-55)	0.503
Respiratory mechanics pre-decannulation†				
Respiratory rate, breaths/min	18 (16−20)	18 (16−20)	17 (16.5−20)	0.112
No accessory resp muscle involvement	40 (91)	76 (93.8)	50 (87.7)	0.356
Accessory muscles involvement (any)	1 (2.3)	4 (5.9)	5 (8.7)	0.356
Sternomastoid	0 (0)	3 (3.7)	3 (5.2)	0.337
Transversus abdominis	1 (2.3)	1 (1.2)	2 (3.5)	0.656
Thoracoabdominal asynchrony	0	0	0	–
Outcomes				
Hospital stay, days	41 (34−49)	65 (54−76)	94 (85−116)	<0.001
Duration of IMV, days	14 (7−19)	13 (6−18)	16 (12−22)	0.107
In-hospital death	0	1 (1.2)	3 (5.0)	0.160
Time to decannulation, days	17 (14−18)	30 (25.5−35.5)	56 (46−75)	<0.001
Time from decannulation to discharge, days	9 (5−17)	13 (8−22)	13 (6−25)	0.212

Early: <20 days; average: 20–40 days; late: >40 days. Blood gas analysis values are referred to the pre-decannulation day. Data are expressed as frequencies (prevalence) or medians (IQR). P-values are 2-sided. Bonferroni correction was applied for multiple comparisons for independent samples in case of non-normally distributed data.

FiO_2_ = inspired fraction of oxygen; IMV = invasive mechanical ventilation; PaO_2_ = arterial partial pressure of oxygen; PaCO2 = arterial partial pressure of carbon dioxide; QsQ score = Quantitative semi Quantitative clinical score.

* Complete data were available in 126 patients.

** Data was available only in 10 patients.

*** Complete data were available in 125 patients.

† Data on accessory respiratory muscle activation was not available in 3 patients (7%) in the early decannulation group, in 1 patient (1.2%) in the average decannulation group and in 6 patients (10.5%) in the late decannulation group.

After adjusting for age, tracheostomy technique, and timing of tracheostomy, changing the caliber of tracheostomy cannula during the weaning process predicted earlier decannulation (OR 4.224, 95%CI: 1.037–17.207; P = 0.044). The assessment of swallowing function was independently associated with slower decannulation (OR 0.161, 95%CI: 0.037−0.694; P = 0.014).

### Feasibility of the QsQ score

**Fig. 2:**
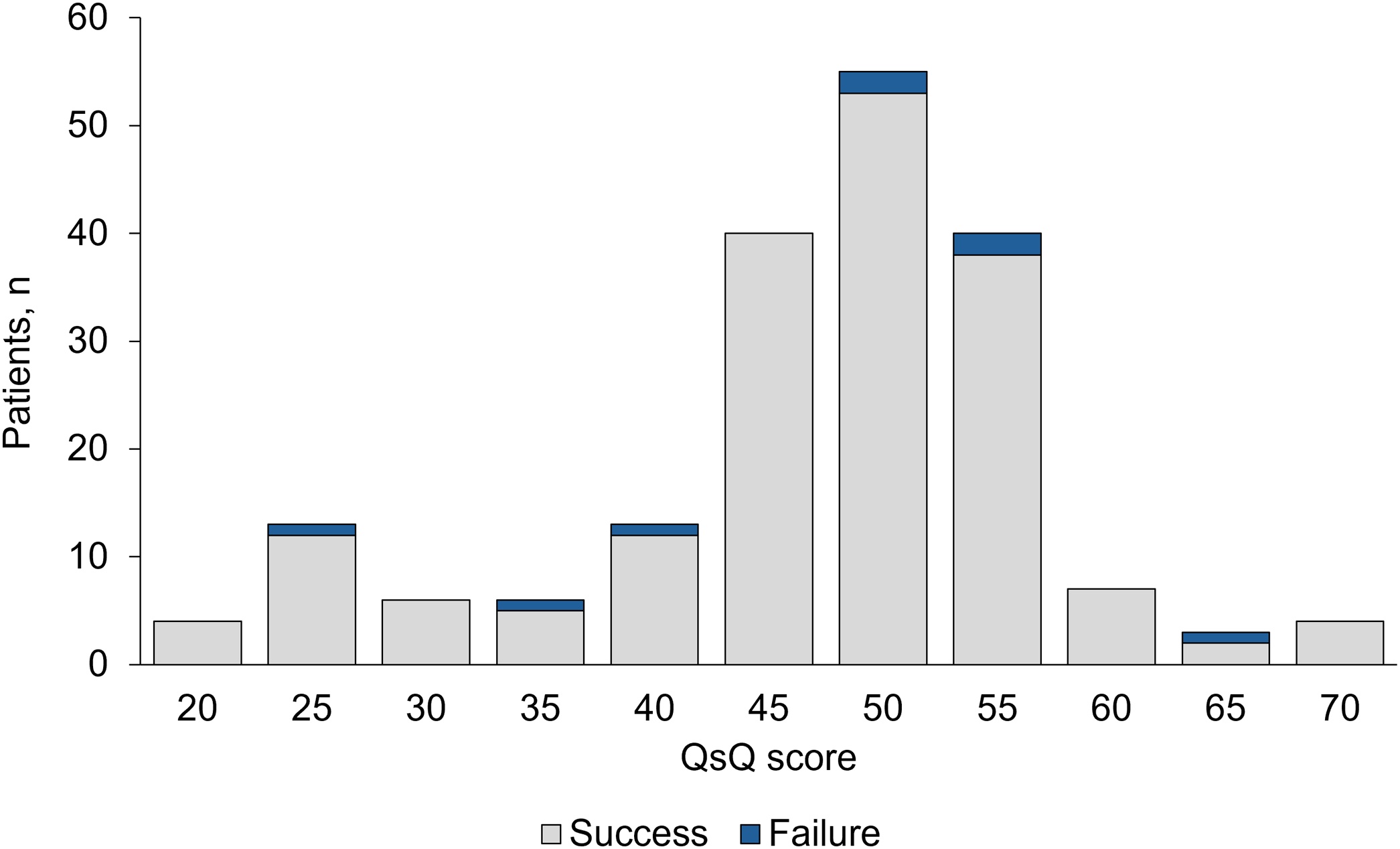
QsQ score distribution in the study cohort. Data are divided by patients that succeeded (light grey) and failed (blue) decannulation. QsQ = Quantitative semi-Quantitative score.

Data and procedures used for QsQ criteria were available for the vast majority of patients, except for the peak cough flow, that was tested in 5.2% of cases (Supplementary Fig. S1). The proportion of satisfied major and minor criteria, for patients with available data, was generally above 80% (Supplementary Fig. S2). QsQ scores were not associated with decannulation failure (Fig. 2) or with weaning duration (Table 3). Eight patients (4.2%) had both major criteria satisfied, while 91.6% of patients had at least 5 minor criteria satisfied (Supplementary Table S6). Sensitivity of QsQ criteria in predicting decannulation success was high, while specificity was low (Supplementary Tables S7–S8).

Within participating center differences in terms of clinical outcomes, time to decannulation and weaning procedures of interest are reported in Supplementary Table S9.

## Discussion

The findings of the study can be summarized as follows: (1) performing bronchoscopy with nasal access is associated with faster decannulation, and earlier decannulation is independently associated with cannula caliber reduction during the weaning process; (2) need to test for swallowing function predicted a longer weaning process; (3) the burden of bronchial secretions appeared the only factor associated with decannulation failure, although failure occurred only in 4.2% of the study cohort; (4) QsQ feasibility was limited, and the score was poorly accurate in predicting decannulation success.

Tracheostomy decannulation failure is generally defined as the need to reinsert the tracheostomy cannula from 48 h up to 3 months after a first decannulation attempt [10]. Indeed, the timing and success rate of such procedure has been reported to be very variable [10], ranging from 23% [18] in studies involving neuromuscular disease and sleep apnoea patients, up to 100% [19]. In our study, the 48 -h threshold to consider decannulation failure was arbitrarily selected, based upon expert opinion and clinical judgement, and reflected the results of the cross-sectional survey carried out in 2008 by Stelfox er al [16], that explored the general acceptance of different time-to-failure definitions. Among the 225 experts interviewed, including physicians and respiratory therapists, the majority of respondents judged the 48-h time frame as the most reasonable threshold to define decannulation failure [16]. To date, however, the ideal time frame to consider decannulation failure remains debated. Large retrospective [20] and prospective [21] observational studies showed that the majority of patients fail decannulation within 48 h. Of interest, in the study by Ghiani and colleagues, 72.5% of patients failed within 48 h, and of those, 48% were re-incannulated in the first 4 h. In our study, decannulation failure occurred in only 8 out of 191 patients, and re-institution of tracheostomy cannula occurred in the first 24 h in the majority of cases (median 12 (12–42) hours, 67% of cases ≤12 h), secondary to the patients’ inability to cope with bronchial secretions, unexpected tracheal granulomas or vocal cord dysfunction, all factors that imply an acute increase of upper and lower airway resistances, consequently causing an increased work of breathing, but also affecting swallowing, cough and favoring aspiration [13]. In the present study only two patients that were considered successfully weaned from tracheostomy were later re-incannulated after an average of 6.5 days from decannulation, in both cases for respiratory failure secondary to hospital acquired pneumonia. As the reason for failure varies with increasing time to re-incannulation (from respiratory distress, respiratory failure, swallowing impairment, to aspiration pneumonia or hospital acquired infections), it would be of undoubted clinical and pathophysiological interest to differentiate between immediate/early and late decannulation failure. Therefore, although the number of potentially mis-classified patients in the present study would be low, in our case the adoption of the 48-h time frame could have biased patient grouping to some extent.

We hypothesize that the reason for a low proportion of patients meeting failure criteria both in our study and in some previous reports [[22], [23], [24], [25], [26], [27]], should be secondary to two main factors: the absence of per-protocol weaning, and a flawed medical perception in judging the readiness of patients to commence weaning or undergo tube capping. The latter would justify the average time to decannulation of >30 days. In fact, tube capping is usually a step that comes after a process that might (heterogeneously) include: separation from mechanical ventilation, clinical stabilization, sufficient bronchial secretion reduction and their satisfactory management by the patient, assessment of cough and swallowing, and also tracheostomy tube downsizing and/or speaking valve placement [8,16]. On the other hand, starting the weaning process implies the decision to reduce or stop the invasive respiratory support or to start decreasing the non-invasive support in a patient with a tracheostomy tube. The former, usually, implies having proceeded with the latter.

Weaning from tracheostomy should parallel the process of weaning from mechanical ventilation, and should contemplate: (1) procedures that establish patient’s clinical stability, (2) decannulation screening/readiness tests and (3) a confirmatory test, which in this case is represented by the decannulation itself.

While in some cases effective decannulation might occur also in absence of endoscopic airway inspection [28], ensuring airway patency and clearance is often advisable to proceed with further weaning steps and confirm patient’s stability. In our cohort, upper and lower airway inspection with bronchoscopy was associated with a shorter time to decannulation. Indeed, tracheal stenosis and a higher secretion burden were observed more frequently in patients with delayed decannulation and were significantly associated with decannulation failure. Accordingly, a progressive reduction of the cannula caliber was the only factor that predicted faster weaning. We speculate that reducing the size of the tracheostomy cannula allows the progressive restoration of physiological breathing and facilitates the detection of factors that may hinder the decannulation process, thus identifying patients less prepared to tolerate tube capping. Moreover, it can be responsible of progressive cough improvement and can aid swallowing rehabilitation, further impacting the decannulation process and weaning outcome [29]. In our case, swallowing assessment prolonged the duration of weaning, probably due to the longer time to organize and perform these tests in clinical practice and to the tendency to perform such tests in patients suspected of having swallowing disorders, as suggested by the trend of moderate-severe dysphagia in patients with longer time to decannulation. In this view, we demonstrated that performing bronchoscopy also with nasal access was associated with lower frequency of swallowing assessments during weaning, which might partly explain why nasal bronchoscopy was associated with shorter weaning duration and further suggesting that upper and lower airway inspection might have reduced the suspect of swallowing disorders. Nevertheless, the advantage of nasal access should be cautiously regarded, as it appeared progressively weaker with longer decannulation time, implying a survival bias affecting patients that tended to be exposed longer to IMV or with more severe swallowing impairment (Fig. 1 and Table 3).

The second stage of weaning should include a screening test in patients with satisfactory clinical stability, i.e. tube capping. Tube capping was performed only in 83% of patients. This confirms the observations by Hernandez Martinez and colleagues, that showed faster decannulation in patients not exposed to tube capping but deemed ready for decannulation based on suctioning frequency (≤2 aspirations every 8 h over a 24 h period) while on high flow nasal cannula (HFNC) support [28]. In the same study, patients that underwent a tube capping trial (i.e. tolerated tube capping ≥24 h) doubled the time to decannulation (median of 6 vs 13 days) and increased length of hospital stay, although decannulation failure was not different between groups [28], leaving the question open if tube capping should be performed per-protocol or avoided in some patients in which a sudden increase in airway resistances can lead to fail this type of screening test. In this context, a short-term capping (minutes or few hours) could be able to identify patients at potential risk of early failure and serve as a surrogate screening test. Nevertheless, the high number of successes, the extensive length of the weaning process, and the frequent satisfaction of major and minor criteria of the QsQ score, indicated that the majority of patients were probably ready for a weaning trial much earlier than observed. In the same time, inspecting upper airways by means of bronchoscopy might have facilitated weaning avoiding decannulation in patients with relevant anatomical or functional alterations of larynx and/or vocal cords.

Physicians are usually slow in recognizing patient readiness to tolerate a spontaneous breathing trial [[30], [31], [32]]. We speculate that this might also be true in tracheostomized patients, suggesting why per-protocol testing for decannulation readiness should be performed as soon as clinical stability is met. Tube capping is associated with variable changes in breathing mechanics, and this could depend upon the size of the cannula, upper and lower airways’ anatomy, presence of respiratory comorbidities, and muscle weakness as a consequence of prolonged ICU stay and upper airway disuse [8]. This also explains why in our study, weaning length was largely independent of the vital signs, accessory respiratory muscle activation, level of consciousness and gas exchange parameters before decannulation. On the other hand, patients that failed decannulation were more likely to show signs of accessory respiratory muscle activation (especially sternomastoid activation) compared with patients that succeeded in the weaning process. Unfortunately, due to the small number of patients that failed, further reasoning on the role of indirect signs of muscle fatigue on the decannulation process would be speculative.

The third stage of weaning is represented by a confirmatory test, the decannulation itself. Due to the very low number of failures, predictors of decannulation failure could not be tested in our study. In this view, the QsQ clinical score results were also very limited by the low number of patients that failed decannulation. We observed an overall failure rate of 4.2%. This result is in line with a large study conducted in Spanish ICUs aimed at investigating if frequency of suctioning rather than tube capping intervaled with HFNC could provide faster decannulation [25]. Hernández Martínez and colleagues observed a decannulation failure that ranged from 5.6% in the control group to 2.4% in the intervention group [28]. In other settings, such as in patients hospitalized in long term weaning centers, a survey highlighted a decannulation success in only 22% of patients [28]. The wide difference in outcomes is justified mainly by different age and inclusion criteria. More than 80% of patients in the study by Hernández Martínez had a trauma or surgical diagnosis at ICU admission and a mean age of ∼58 years old [28], while Marchese et al. recruited patients that were >70 years old in 61.6% of cases and almost one-third were patients with a neuromuscular disorder [18]. In our study we excluded patients with known neuromuscular disease and also patients with acute brain injury, including stroke, traumatic brain injury, encephalopathy complicating cardiac arrest. In terms of QsQ feasibility, >90% of patients had the necessary data to test criteria, and 7 out of 10 criteria were available in >98% of cases. However, peak cough flow was almost never performed during the weaning process. As expected, the score returned very high sensitivity values, meaning that was able to discern patients that will be successful in the decannulation process. Accordingly, due to a spectrum and test referral bias [30], specificity was very low. In fact, as our variable of interest was tolerance of decannulation, and decannulation was used to prove the reliability of weaning predictors such as tube capping (performed in the majority of cases of our cohort), the need of passing a tube capping trial might have excluded patients that did not pass such procedure during the hospital stay, and therefore never underwent a decannulation trial. Consequently, the study cohort was probably skewed towards less severe patients, affecting sensitivity and specificity of the QsQ score. Indeed, the proposed score threshold of 40 points in the original proposal was not suitable to detect decannulation outcomes, and some of the QsQ criteria appeared to have either poor significance in the weaning process (e.g. age or comorbidities), or were not systematically tested (such as the peak cough flow, swallowing or capping failure) [8], further significantly affecting QsQ results and any clinical conclusion about its predictivity in this specific cohort. In particular, peak cough flow was available in only 10 out of 191 patients, with no relationship with decannulation outcome, suggesting the rationale for restricting the number of criteria and questioning the strength of such variable as a predictor of decannulation weaning outcome in clinical practice. Prospective specifically designed studies should explore the added value of each criterion and their correlation with decannulation.

### Study limitations

The study has several limitations. The results suffer from a selection bias, having excluded brain injured, post-trauma or patients with chronic neuromuscular disease, moreover, two thirds of the pneumonia cases were due to COVID-19, affecting overall generalizability. Indeed, the study period was characterized by a high prevalence of patients with COVID-19, explaining the relatively low prevalence of comorbidities in the study cohort, that might have influenced patients’ complexity, clinical severity, and study outcomes, especially weaning duration. Despite it might have represented a factor influencing the time to decannulation, ICU-required weakness and its severity were not assessed. This was mainly due to the study design and the heterogeneity of ICU-required weakness diagnosis among centers. The decision to exclude patients that did not undergo a tracheostomy weaning trial had undoubtedly impacted the failure rate, but it would be less informative in terms of weaning procedures and time-to-wean predictors, representing, due to the observational and not per-protocol design, a substantial selection and non-response bias. Time-lags used to group patients into early, average and late decannulation were operational and therefore might have further limited the generalizability of the results. Weaning protocols were heterogenous among participating centers, affecting type and timing of different weaning steps (e.g. peak cough flow), time to decannulation and overall interpretation of the QsQ results. Tube capping failure was not collected, therefore the sensitivity and specificity of this test could not be calculated. We acknowledge that inferential statistics for decannulation failure should be considered with caution. In fact, the numerosity of the failure group could have been underestimated by the time frame chosen to define decannulation failure, and by the sample size. Neverthless, only two cases needed re-incannulation after ≥48 h post-decannulation, and, to the best of our knowledge, this represents one of the largest cohort studies performed in patients undergoing weaning from tracheostomy.

## Conclusions

This large retrospective study showed that specific procedures such as bronchoscopy and reduction of the cannula caliber are associated with shorter time to decannulation. However, the overall significant length of the weaning process and the low proportion of decannulation failures suggest the absence of per-protocol weaning practices and possible procrastination in performing weaning readiness screening tests.

Research priority should be given to the identification of early physiological predictors of screening failure during weaning from tracheostomy and to randomized trials designed to test different per-protocol strategies in patients undergoing a tracheostomy weaning process.

## CRediT authorship contribution statement

Concept and Design: DR and PS; Data acquisition and analysis: DR, MG, JCS, PS; Data interpretation: DR, FDM, MM, CC, AG, MG, JCS, FR, CA, GM, FBAB, PS; Writing the manuscript draft: DR; FDM, MM, CC, AG, PS; Supervision: PS, FDM, MM, CC, FBAB, and PS; Visualization: DR, MG, JCS; All Authors critically revised and approved the final version of the manuscript.

## Consent for publication

Not applicable

## Ethics approval and consent to participate

The study was conducted in accordance with the amended declaration of Helsinki and was approved by the local Ethics Committee (Comitato Etico Area 1 - 2022/ST/061).

## Funding

This research did not receive any specific grant from funding agencies in the public, commercial, or not-for-profit sectors.

## Availability of data and materials

The datasets generated and analyzed during the current study will be made available by the Corresponding Authors upon reasonable request.

## Declaration of competing interest

The Authors have no conflicts of interest to declare pertaining to the submitted manuscript. Competing interests outside the submitted work: DR received honoraria for lectures from Astra Zeneca, Berlin Chemie, Glaxo Smith Kline, Menarini Farmaceutici, Roche, Sanofi, Zambon, and fees for consultancy from Astra Zeneca, Insmed, Sanofi; FDM has received honoraria for lectures, served as a consultant, and received financial support for research funds and fees from AstraZeneca, Boehringer Ingelheim, Novartis, Pfizer, Chiesi Farmaceutici, Guidotti/Malesci, Glaxo Smith Kline, and fees for advisory boards, consultation, education and presentations from Austral, Biosency, MSD, AstraZeneca, Chiesi, Menarini, Nuvaira, Neopharmed Gentili, Novartis, Sanofi and Zambon; CC received honoraria for lectures from Fisher & Paykel, Philips, ResMed, Vitalair, Medicair, and fees for advisory boards from Airliquide; AG received honoraria for lectures from Astra Zeneca, Insmed, Vertex and Zambon, and consulting fees from Insmed and Boehringer Ingelheim; FBAB received honoraria for lectures from Astra Zeneca, GlaxoSmithKline, Insmed Inc., Menarini International, OM Pharma, Pfizer, Sanofi Pharmaceuticals, Vertex Pharmaceuticals, Zambon, and fees for consultancy from Chiesi and OM Pharma; PS received honoraria for lectures from AstraZeneca, Berlin-Chemie, GlaxoSmithKline, Gilead Sciences and Sanofi, grants or contracts from AstraZeneca, Edmond Pharma, GlaxoSmithKline and Novartis, consultancy fees from AstraZeneca, Berlin-Chemie, Bruschettini, Dompé Farmaceutici, Edmond Pharma, GlaxoSmithKline, Neopharmed Gentili and Sanofi. MM, MG, JCS, FR, CA, GM have no competing interests to disclose outside the submitted work.

## Glossary

COVID-19: Coronavirus disease 2019
ETI: endotracheal intubation
GCS: Glasgow coma scale
ICU: Intensive Care Unit
IQR: inter-quartile range
IMV: invasive mechanical ventilation
OR: odds ratio
PaO_2_: arterial partial pressure of oxygen
QsQ: Quantitative semi-Quantitative clinical score
