# Supplementary material for: Determinants of time to decannulation and predictors of early weaning from tracheostomy: a multicenter, retrospective Italian cohort study
Source: Ann Intensive Care. 2026 Mar 17;16:100050. doi: 10.1016/j.aicoj.2026.100050 (PMC13011248; doi:10.1016/j.aicoj.2026.100050)
Supplement: Supplementary file 1 [file mmc1.docx]

**Supplementary Information File**

**Title**

Determinants of time to decannulation and predictors of early weaning from tracheostomy: a multicenter, retrospective Italian cohort study.

**Authors**

Dejan Radovanovic^1,2,3^, Fabiano Di Marco^4,5^, Michele Mondoni^6^, Claudia Crimi^7,8^, Andrea Gramegna^9,10^, Marina Gatti^2^, Juan Camilo Signorello^2^, Federico Raimondi^5^, Cristina Albrici^6^, Giorgio Morana^8^, Francesco Bruno Arturo Blasi^9,10^, Pierachille Santus^1,2,3^*

*** Corresponding Author**

Pierachille Santus, MD, PhD Division of Respiratory Diseases, Department of Biomedical and Clinical Sciences (DIBIC), Università di Milano, Ospedale L. Sacco, ASST Fatebenefratelli-Sacco, Via G.B. Grassi 74, 20157 Milano, Italy. Tel. +39.02.39042801 – Fax: +39.02.39042473. E-mail: [pierachille.santus@unimi.it](mailto:pierachille.santus@unimi.it)

**Affiliations**

^1^ Department of Biomedical and Clinical Sciences (DIBIC), Università degli Studi di Milano; ^2^ Division of Respiratory Diseases, Ospedale L. Sacco, ASST Fatebenefratelli-Sacco, Milano, Italy; ^3^ Coordinated Research Center on Respiratory Failure, University of Milan, Milano, Italy; ^4^Department of Health Sciences, Università Degli Studi Di Milano, Milan, Italy; ^5^ Respiratory Unit, ASST Papa Giovanni XXIII Hospital, Bergamo, Italy; ^6^ Respiratory Unit, Department of Health Sciences, ASST Santi Paolo e Carlo, Università degli Studi di Milano, 20145 Milan, Italy; ^7^ Department of Clinical and Experimental Medicine, University of Catania, Catania, Italy; ^8^ Respiratory Intensive Care Unit, Respiratory Medicine Unit, Policlinico "G. Rodolico-San Marco" University Hospital, Catania, Italy; ^9^ Department of Pathophysiology and Transplantation, Università degli Studi di Milano; ^10^ Respiratory and Cystic Fibrosis Unit, Fondazione IRCCS Ca’ Granda Ospedale Maggiore Policlinico Milano, Milano, Italy.

**Supplementray Material and Methods**

***QsQ score***

The QsQ score has been proposed by Santus et al to simplify the identification of patients with a higher chance to be weaned from tracheostomy cannula (*Santus P, Gramegna A, Radovanovic D, Raccanelli R, Valenti V, Rabbiosi D, et al. A systematic review on tracheostomy decannulation: a proposal of a quantitative semiquantitative clinical score. BMC Pulm Med 2014;****15****:201. https://doi.10.1186/1471-2466-14-2*). The score was based upon a critical research of literature, and included two major and eight minor criteria clinical criteria among those more frequently proposed as weaning predictors (Supplementary Table 1). The Authors suggest that a positive decannulation could be expected if the following criteria combination were met: two major criteria, eight minor criteria, or a major criterion in addition to 5/8 minor criteria. A cutoff score of ≥40 points was proposed to predict successful decannulation. For the present study, availability and satisfaction of the QsQ criteria were assessed and the total score computed.

**Supplementary Results**

***Reason for patients’ exclusion***

Of the 196 patients enrolled, three patients were excluded because they met *a-posteriori* the exclusion criteria (two self-decannulations and one patient with a history of amyotrophic lateral sclerosis), and two patients because of incomplete data.

***Decannulation failure***

Decannulation failure was due to respiratory distress (n = 4), tracheal granuloma (n = 3) and vocal cord paralysis (n = 1). The median (inter-quartile range) time to re-incannulation was 12 (12-42) hours; in six cases, re-incannulation occurred in the first 12 hours post decannulation. Two patients that succeeded decannulation, were later re-incannulated (one after 7 days, one after 6 days from decannulation) because of acute type II respiratory failure due to hospital acquired pneumonia. According to the study protocol and design, these patients were not excluded from the study.

| **Quantitative parameters – main (major) criteria** | | |
| --- | --- | --- |
| Cough assessment | MEP ≥ 40 cmH2O, or  PCF > 160 l/min | 20 points |
|  |  |  |
| Tube capping | ≥ 24 hours | 20 points |
| **Semi-quantitative parameters – minor criteria** | | |
| Consciousness | Drowsy vs alert | 5 points |
| Secretions | Thick vs thin | 5 points |
| Swallowing | Impaired vs Normal | 5 points |
| PaCO2 | < 60 mmHg | 5 points |
| Airways | Tracheal stenosis <50% | 5 points |
| Age | < 70 years | 5 points |
| Indication for tracheostomy | Pneumonia or airway obstruction vs others | 5 points |
| Comorbidities | None vs ≥ 1 | 5 points |

**Supplementary Table 1**. QsQ clinical score major and minor criteria. Points are assigned if the criterion is assessed and: the patient has a MEP ≥ 40 cmH2O or a peak cough flow > 160 l/min (20 points), met or exceeded ≥24 hours of tube capping (20 points), is alert (5 points), has any impairment in swallowing (5 points), has a PaCO2 <60 mmHg (5 points), has a tracheal stenosis <50% of tracheal lumen assessed with bronchoscopy (5 points), is <70 years old (5 points), underwent tracheostomy because of pneumonia or upper airway obstruction (5 points), has no comorbidities (5 points). A high probability of positive decannulation has been proposed if a patients meets: both major criteria, one major criterion and at least >50% of minor criteria, all minor criteria irrespective of the major criteria. Modified from: *Santus P, Gramegna A, Radovanovic D, Raccanelli R, Valenti V, Rabbiosi D, Vitacca M, Nava S (2014). A systematic review on tracheostomy decannulation: a proposal of a quantitative semiquantitative clinical score. BMC Pulm Med 15;14:201.* [*https://doi.10.1186/1471-2466-14-2*](https://doi.10.1186/1471-2466-14-2).. MEP = maximal expiratory pressure; PCF = peak cough flow. PaCO2 = arterial partial pressure of carbon dioxide.

| **Comorbiditiy** | **All** | **Success** | **Failure** | **p-value** |
| --- | --- | --- | --- | --- |
|  | **N = 191** | **N = 183** | **N = 8** |  |
| ≥ 1 comorbidity, n (%) | 152 (79.6) | 146 (79.8) | 6 (75) | 0.7182 |
| Obesity, n (%) | 32 (16.8) | 30 (16.4) | 2 (25) | 0.623 |
| Diabetes, n (%) | 41 (21.5) | 39 (21.3) | 2 (25) | 0.682 |
| Diabetes type II, n (%) | 31 (16.2) | 30 (16.4) | 1 (12.5) | 0.629 |
| Diabetes type I, n (%) | 10 (5.2) | 9 (4.9) | 1 (12.5) |  |
| Ischemic heart disease, n (%) | 29 (15.2) | 28 (15.3) | 1 (12.5) | 0.651 |
| Chronic heart failure, n (%) | 18 (9.4) | 17 (9.2) | 1 (12.5) | 0.546 |
| Reduced ejection fraction, n (%) | 11 (5.7) | 10 (5.5) | 1 (12.5) | 0.468 |
| Preserved ejection fraction, n (%) | 7 (3.6) | 7 (3.7) | 0 | - |
| COPD, n (%) | 25 (13.1) | 23 (12.6) | 2 (25) | 0.281 |
| Solid tumors, n (%) | 16 (8.4) | 15 (8.2) | 1 (12.5) | 0.510 |
| Neurological disease*, n (%) | 14 (7.3) | 14 (7.7) | 0 | - |
| Rheumatological disorder, n (%) | 10 (5.2) | 10 (5.5) | 0 | - |
| Cerebrovascular disease, n (%) | 9 (4.7) | 8 (4.7) | 1 (12.5) | 0.325 |
| Chronic kidney disease, n (%) | 8 (4.2) | 8 (4.2) | 0 | - |
| Hematological disease, n (%) | 8 (4.2) | 8 (4.2) | 0 | - |
| Gastrointestinal disease, n (%) | 8 (4.2) | 7 (4.2) | 1 (12.5) | - |
| Liver disease, n (%) | 8 (4.2) | 8 (4.2) | 0 | - |
| Lymphoproliferative disorder, n (%) | 7 (3.7) | 6 (3.7) | 1 (12.5) | - |
| Asthma, n (%) | 7 (3.7) | 7 (3.7) | 0 | - |
| Pulmonary fibrosis, n (%) | 6 (3.1) | 3 (3.1) | 3 (37.5) | - |
| Sleep apnoea, n (%) | 6 (3.1) | 6 (3.1) | 0 | - |
| Atrial fibrillation, n (%) | 5 (2.6) | 5 (2.6) | 0 | - |
| Psychiatric disorder. n (%) | 4 (2.1) | 4 (2.1) | 0 | - |
| Organ transplant, n (%) | 4 (2.1) | 4 (2.1) | 0 | - |
| Immune depression, n (%) | 3 (1.6) | 3 (1.6) | 0 | - |
| Pulmonary hypertension, n (%) | 1 (0.5) | 1 (0.5) | 0 | - |
| Pulmonary embolism, n (%) | 1 (0.5) | 1 (0.5) | 0 | - |
|  |  |  |  |  |

**Supplementary Table 2**. List of comorbidities of the study cohort and in patients that succeeded or failed decannulation. Data are reported as frequencies and prevalence. COPD = Chronic Obstructive Pulmonary Disease. * Neurological disease included a positive history of any of the following: Parkinson disease, Alzheimer’s disease, Parkinson’s disease, essential tremor, epilepsy, chronic migraine.

| **Diagnosis at ETI** | **All** | **Success** | **Failure** | **p-value** |
| --- | --- | --- | --- | --- |
|  | **N = 191** | **N = 183** | **N = 8** |  |
| **Pneumonia** | 151 (79.1) | 146 (79.8) | 5 (62.5) | 0.367 |
| ***Community Acquired*** | 142 (74.3) | 137 (74.9) | 5 (62.5) | 0.425 |
| SARS-CoV-2 | 117 (61.2) | 112 (61.2) | 5 (62.5) | 1.000 |
| Pneumococcus pneumoniae | 15 (7.8) | 15 (8.2) | 0 | 1.000 |
| Influenza virus A | 6 (3.1) | 6 (3.3) | 0 | 1.000 |
| Aspiration | 3 (1.6) | 3 (1.6) | 0 | 1.000 |
| Other causes | 1 (0.5) | 1 (0.5) | 0 | - |
| ***Hospital Acquired*** | 9 (4.7) | 9 (4.9) | 0 | 1.000 |
|  |  |  |  |  |
| **Shock** | 24 (12.6) | 22 (12.0) | 2 (25.0) | 0.265 |
| Cardiogenic | 13 (6.8) | 11 (6.0) | 2 (25.0) | 0.095 |
| Septic | 11 (5.8) | 11 (6.0) | 0 | 1.000 |
|  |  |  |  |  |
| **Obstructive disease exacerbation** | 5 (2.6) | 5 (2.7) | 0 | 1.000 |
| COPD Exacerbation | 4 (2.1) | 3 (0.5) | 1 (12.5) | 0.158 |
| Asthma Exacerbation | 1 (0.5) | 1 (0.5) | 0 | - |
|  |  |  |  |  |
| **Other cause** | 11 (5.8) | 11 (6.0) | 0 | 1.000 |
| Aortic dissection type A | 3 (1.6) | 3 (1.6) | 0 | 1.000 |
| Meningitis | 5 (2.6) | 5 (2.7) | 0 | 1.000 |
| Pneumothorax | 1 (0.5) | 1 (0.5) | 0 | - |
| IPF | 1 (0.5) | 1 (0.5) | 0 | - |
| Tetanus | 1 (0.5) | 1 (0.5) | 0 | - |
|  |  |  |  |  |
|  |  |  |  |  |

**Supplementary Table 3.** Diagnoses that led to endotracheal intubation in the study cohort and in patients that succeeded and failed the weaning from tracheostomy. Data are presented as frequencies (prevalence). COPD = Chronic Obstructive Pulmonary Disease; IPF = Idiopathic Pulmonary Fibrosis. * Legionella pneumophila

| **Variable** | **All** | **Success** | **Failure** | **p-value** |
| --- | --- | --- | --- | --- |
|  | **N = 191** | **N = 183** | **N = 8** |  |
| Cough efficiency | 176 (92.1) | 169 (92.3) | 7 (87.5) | 0.487 |
| Swallowing assessment | 128 (67) | 121 (66.1) | 7 (87.5) | 0.195 |
| *Swallowing* | | | |  |
| Normal, n (%) | 82 (64.6) | 77 (42.1) | 5 (62.5) | 0.706 |
| Mild impairment, n (%) | 26 (20.5) | 24 (13.1) | 2 (25) |  |
| Moderate/severe impairment, n (%) | 19 (14.5) | 19 (9.8) | - |  |
| Efficient cough, n (%) | 176 (92.1) | 169 (92.3) | 7 (87.5) | 0.487 |
|  |  |  |  |  |
| *Bronchoscopy* | | | |  |
| Not performed | 45 (24%) | 44 (23) | 1 (12.5) | 0.363 |
| Tracheostomy access, n (%) | 54 (28.3) | 50 (27.3) | 4 (50) |  |
| Nasal access, n (%) | 92 (48.2) | 89 (48.6) | 3 (37.5) |  |
| Tracheal stenosis, n (%) | 11 (5.9) | 9 (4.9) | 2 (25) | 0.072 |
|  |  |  |  |  |
| *Tracheostomy cannula characteristics* |  |  |  |  |
| Cannula caliber* |  |  |  |  |
| ID 4 mm, n (%) | 6 (4.8) | 5 (4.2) | 1 (20) | 0.421 |
| ID 5 mm, n (%) | 0 | - | - |  |
| ID 6 mm, n (%) | 16 (12.9) | 16 (13.4) | 0 |  |
| ID 7 mm, n (%) | 12 (9.7) | 11 (9.2) | 1 (20) |  |
| ID 8 mm, n (%) | 88 (71) | 85 (71.4) | 3 (60) |  |
| ID 9 mm, n (%) | 2 (1.6) | 2 (1.7) | 0 |  |
| Cuff present** | 86 (89.6) | 82 (89.1) | 4 (100) | 0.486 |
| Cuff absent** | 10 (10.4) | 10 (10.9) | 0 |  |
| Fenestrated*^†^* | 19 (63.3) | 17 (60.7) | 2 (100) | 0.265 |
| Not fenestrated*^†^* | 11 (36.7) | 11 (39.2) | 0 |  |
| Cannula caliber reduction, n (%) | 90 (47.1) | 88 (48.1) | 2 (25) | 0.180 |
| Tube capping, n (%) | 160 (83.8) | 154 (84.2) | 6 (75) | 0.383 |
| *Respiratory support post decannulation^††^* | | | | |
| Room air, n (%) | 89 (46.8) | 84 (46.1) | 5 (55.5) | 0.365 |
| Nasal cannula, n (%) | 80 (42.1) | 78 (42.8) | 2 (22.2) | 0.331 |
| HFNC, n (%) | 13 (6.8) | 12 (6.5) | 1 (11.1) | 0.517 |
| Venturi mask, n (%) | 7 (3.6) | 7 (3.8) | 0 | 0.572 |
| Non-invasive ventilation, n (%) | 1 (0.5) | 1 (0.5) | 0 | 0.834 |
| *Outcomes* |  |  |  |  |
| Decannulation failure, n (%) | 8 (4.2) | - | - | - |
| Time to decannulation, n (%) | 31.5 (21-46) | 31.5 (21-74) | 34 (18-76) | 0.760 |
| Lenght of stay, days | 65 (48-91) | 66 (48-91) | 62 (46-118) | 0.825 |
| In-hospital mortality, n (%) | 4 (2.1) | 3 (1.6) | 1 (12.5) | 0.159 |
|  |  |  |  |  |
|  |  |  |  |  |

**Supplementary Table 4.** **Procedures and tracheostomy-related characteristics and outcomes.** Data are reported in patients that succeeded and failed decannulation. * data were available for 124 patients (n = 5 failures); ** data were available for 96 patients (n = 4 failures); ^†^ data were available for 30 patients (n = 2 failures); ^††^ data were available for 190 patients (n = 9 failures). ID = inner diameter; HFNC = high flow nasal cannulae.

|  | **All N = 191** | | **Success (A) N = 183** | | **Failure (B) N = 8** | |  |
| --- | --- | --- | --- | --- | --- | --- | --- |
| **Variable** | **Valid cases** | **value** | **Valid cases** | **value** | **Valid cases** | **value** | **p-value (A vs B)** |
| *Blood biomarkers* |  |  |  |  |  |  |  |
| WBC, x10^3^ cells/µl | 171 (89.5) | 7000 (5340-8760) | 164 (89.6) | 6995 (5370-8760) | 7 (87.5) | 7010 (3900-9720) | 0.743 |
| Hemoglobin | 176 (92.1) | 10.1 (9.4-11.0) | 169 (92.3) | 10.1 (9.4-10.9) | 7 (87.5) | 10.7 (9.5-12) | 0.550 |
| INR | 115 (60.2) | 1.16 (1.09-1.25) | 109 (59.6) | 1.16 (1.1-1.23) | 6 (75) | 1.19 (1.0-1.53) | 0.568 |
| CRP | 166 (86.9) | 10.1 (2.9-30.5) | 159 (86.9) | 10 (3-30) | 7 (87.5) | 14.4 (1-72) | 0.865 |
| *Gas exchange* |  |  |  |  |  |  |  |
| PaO2, mmHg | 125 (65.8) | 79 (69-96) | 119 (65) | 80 (70-97) | 6 (75) | 67 (60-93) | 0.232 |
| PaCO2, mmHg | 127 (66.8) | 39 (36-42) | 121 (66.1) | 39 (36-42) | 6 (75) | 40 (36-40) | 0.779 |
| pH | 126 (66.3) | 7.45 (7.43-7.48) | 120 (65.6) | 7.45 (7.43-7.48) | 6 (75) | 7.46 (7.44-7-47) | 0.676 |
| FiO2, % | 185 (97.4) | 24 (21-28) | 177 (96.7) | 24 (21-29) | 8 (100) | 21 (21-24) | 0.411 |
| SpO2, % | 175 (92) | 97 (95-98) | 168 (91.8) | 97 (96-98) | 7 (87.5) | 95 (93-99) | 0.962 |
| *Vital signs* |  |  |  |  |  |  |  |
| GCS | 142 (74.7) | 15 (15-15) | 134 (73.2) | 15 (15-15) | 8 (100) | 15 (15-15) | - |
| Temperature, °C | 169 (88.9) | 36.2 (36.0-36.5) | 162 (88.5) | 36.2 (36.0-36.5) | 7 (87.5) | 36 (36-36) | 0.615 |
| Heart rate, beats/min | 152 (80) | 82.7 (13.1) | 163 (89.1) | 83 (75-90) | 7 (87.5) | 79 (64-106) | 0.975 |
| Respiratory rate, breaths/min | 152 (80) | 18 (16-20) | 146 (79.8) | 18 (16-20) | 6 (75) | 20 (19-21) | 0.103 |
| No accessory resp muscle involvement | 181 (94.7) | 171 (94.5) | 172 (94.0) | 163 (94.8) | 8 (100) | 5 (62.5) | **0.015** |
| Accessory muscles involvement (any), n (%) | 181 (94.7) | 10 (5.2) | 172 (93.9) | 7 (4.1) | 8 (100) | 3 (37.5) | **<0.001** |
| Sternomastoid, n (%) | 181 (94.7) | 6 (3.3) | 172 (93.9) | 3 (1.7) | 8 (100) | 3 (37.5) | **<0.001** |
| Transversus abdominis, n (%) | 181 (94.7) | 4 (2.2) | 172 (93.9) | 4 (2.3) | 8 (100) | (0) | 0.671 |
| Thoracoabdominal asynchrony, n (%) | 181 (94.7) | 0 (0) | 172 (93.9) | 0 (0) | 8 (100) | (0) | - |
|  |  |  |  |  |  |  |  |
|  |  |  |  |  |  |  |  |

**Supplementary Table 5.** Blood test, gas exchange parameters, vital signs and signs of respiratory muscle activation collected in the 24 hours before decannulation. Valid cases and means (standard deviation) or medians (inter quartile range) for each variable are presented for the whole study cohort and in patients that succeeded and failed decannulation. Values in bold are statistically significant. CRP = C reactive protein; FiO2 = Fraction of Inspired Oxygen; GCS = Glasgow Coma Scale; INR = International normalized ratio; PaO2 = Arterial partial pressure of oxygen; PaCO2 = Arterial partial pressure of carbon dioxide; SpO2 = Peripheral Oxygen Saturation; WBC = White blood cell count.

|  | **All n = 191** | **Success n = 183** | **Failure n = 8** |
| --- | --- | --- | --- |
| Patients with both major criteria | 8 (4.2) | 7 (3.8) | 1 (12.5) |
| Patients without any major criterion | 31 (16.2) | 29 (15.8) | 2 (25) |
| Patients with 0 minor criteria | 0 | 0 | 0 |
| Patients with ≥ 1 minor criteria | 191 (100) | 183 (100) | 8 (100) |
| Patients with ≥ 2 minor criteria | 191 (100) | 183 (100) | 8 (100) |
| Patients with ≥ 3 minor criteria | 191 (100) | 183 (100) | 8 (100) |
| Patients with ≥ 4 minor criteria | 191 (100) | 183 (100) | 8 (100) |
| Patients with ≥ 5 minor criteria | 175 (91.6) | 168 (91.8) | 7 (87.5) |
| Patients with ≥ 6 minor criteria | 119 (62.3) | 114 (62.3) | 5 (62.5) |
| Patients with ≥ 7 minor criteria | 54 (28.3) | 51 (27.9) | 3 (37.5) |
| Patients with all minor criteria | 0 (0) | 0 (0) | 0 (0) |

**Supplementary Table 6.** Frequency and proportion of patients satisfying all or some major or minor QsQ clinical score criteria within the study cohort and in patients that succeeded or failed decannulation.

| **QsQ parameters** | |  | **Patients that succeeded**  **(n = 183)** | | **Patients that failed**  **(n = 8)** | |
| --- | --- | --- | --- | --- | --- | --- |
| **Major criteria** | Available data for criterion | Criterion satisfied | Succeeded without criterion | Succeeded with criterion | Failedwithout criterion | Failed  with criterion |
| Peak cough flow | 10 (5.2) | 8/10 (80) | 1 (12.5) | 7 (87.5) | 1 (50)* | 1 (50)* |
| Tube capping | 191 (100) | 160/191 (83.8) | 29 (15.8) | 154 (84.2) | 2 (25) | 6 (75) |
| **Minor criteria** |  |  |  |  |  |  |
| Consciousness | 142 (74.3) | 140/142 (98.6) | 1 (0.7) | 133 (99.3) | 1 (12.5) | 7 (87.5) |
| Secretions | 189 (99) | 154/189 (81.5) | 30 (16.6) | 151 (83.4) | 5 (62.5) | 3 (37.5) |
| Swallowing | 127 (66.5) | 108/127 (85) | 19 (15.8) | 101 (84.2) | 0 (0)** | 7 (100)** |
| PaCO2 < 60 mmHg | 191 (100) | 125/191 (98.4) | 2 (1.7) | 119 (98.3) | 0 (0)† | 6 (100)† |
| Tracheal stenosis | 188 (98.4) | 177/188 (94.1) | 9 (5.0) | 171 (95.0) | 2 (25) | 6 (75) |
| Age < 70 years | 191 (100) | 48/191 (25.1) | 137 (74.9) | 46 (25.1) | 6 (75) | 2 (25) |
| Indication for tracheostomy | 191 (100) | 184/191 (96.8) | 6 (3.3) | 176 (96.7) | 0 (0) | 8 (100) |
| Comorbidities (≥ 1) | 191 (100) | 184/191 (96.8) | 7 (3.8) | 176 (92.1) | 0 (0) | 8 (100) |

**Supplementary Table 7.** QsQ score major and minor criteria available for the study cohort, and patients that satisfied criteria in patients that failed and succeeded in the decannulation process. Data are presented as frequencies and prevalence (percentages). * peak cough flow was available only for 2 patients that failed the decannulation process; ** swallowing was available for 7 patients in the decannulation failure group; † PaCO2 was available for 6 patients in the decannulation failure group.

| **Major criteria** | Sensitivity (%) | Specificity (%) | PPV (%) | NPV (%) |
| --- | --- | --- | --- | --- |
| Peak cough flow | 87.5 | 50.0 | 87.5 | 50.0 |
| Tube capping | 84.2 | 25.0 | 96.3 | 6.5 |
| **Minor criteria** |  |  |  |  |
| Consciousness | 99.3 | 12.5 | 95.0 | 50.0 |
| Secretions | 83.4 | 62.5 | 98.1 | 14.3 |
| Swallowing | 84.2 | 0.0 | 93.5 | 0.0 |
| PaCO2 ≤60 mmHg | 98.3 | 0.0 | 95.2 | 0.0 |
| Tracheal stenosis | 95.0 | 25.0 | 96.6 | 18.2 |
| Age < 70 years | 25.1 | 75.0 | 95.8 | 4.2 |
| Indication for tracheostomy | 96.7 | 0.0 | 95.7 | 0.0 |
| Comorbidities (≥ 1) | 96.2 | 0.0 | 95.7 | 0.0 |

**Supplementary Table 8.** Sensitivity, specificity, positive and negative predictive value of QsQ major and negative values. Variables for the prediction of decannulation failure. PPV = positive predictive value; NPV = negative predictive value.

|  | L. Sacco Hospital Milano | S. Paolo Hospital Milano | Policlinico  Ca’ Granda Milano | Papa Giovanni XXIII Bergamo | G. Rodolico S. Marco Catania | P-value |
| --- | --- | --- | --- | --- | --- | --- |
|  | N = 52 | N = 42 | N = 8 | N = 73 | N = 16 |  |
| *Patient characteristics* |  |  |  |  |  |  |
| Males | 12 (23.1) | 12 (28.6) | 3 (37.5) | 18 (24.7) | 6 (37.5) | 0.734 |
| Age, years | 67 (57-73) | 62 (56-70) | 62 (59-72) | 63 (53-70) | 46 (36-61) | <0.001 |
| Arterial hypertension | 30 (57.7) | 16 (38.1) | 6 (75) | 42 (57.5) | 7 (43.8) | 0.141 |
| Ischemic heart disease | 7 (13.5) | 6 (14.3) | 2 (25) | 11 (15.1) | 3 (18.8) | 0.924 |
| Type I diabetes | 4 (7.7) | 4 (9.5) | 0 | 0 | 2 (12.5) | 0.590 |
| Type II diabetes | 6 (11.5) | 6 (14.3) | 1 (12.5) | 18 (24.7) | 0 |  |
| COPD | 6 (11.5) | 3 (7.1) | 1 (12.5) | 12 (16.4) | 3 (18.8) | 0.629 |
| Heart failure | 5 (7.7) | 3 (7.1) | 1 (12.5) | 6 (8.2) | 3 (18.8) | 0.717 |
| Preserved ejection fraction | 2 (3.8) | 1 (2.4) | 0 | 2 (2.7) | 2 (12.5) | 0.740 |
| Reduced ejection fraction | 3 (5.8) | 2 (4.8) | 1 (12.5) | 4 (5.8) | 1 (6.2) | 0.943 |
| *Main diagnosis* |  |  |  |  |  |  |
| Pneumonia | 46 (90.2) | 37 (25.3) | 4 (2.7) | 51 (34.9) | 8 (66.7) | 0.010 |
| COVID-19 | 40 (78.4) | 18 (43.9) | 3 (42.9) | 43 (59.7) | 8 (66.7) | 0.013 |
| Shock | 2 (3.8) | 3 (7.1) | 1 (12.5) | 14 (19.4) | 2 (12.5) | 0.090 |
| *Weaning procedures* | | | | | | |
| Tube capping | 45 (86.5) | 38 (90.5) | 5 (62.5) | 56 (76.7) | 16 (100) | 0.038 |
| Cannula Caliber reduction | 33 (63.5) | 39 (92.9) | 2 (25.0) | 16 (21.9) | 0 | <0.001 |
| Swallowing assessment | 46 (88.5) | 6 (14.3) | 6 (75) | 55 (75.3) | 15 (93.8) | <0.001 |
| Nasal bronchoscopy | 0 | 38 (90.5) | 2 (25) | 52 (71.2) | 0 | <0.001 |
| Tracheostomy bronchoscopy | 29 (55.8) | 1 (2.4) | 2 (25) | 6 (8.2) | 16 (100) |  |
| QsQ score | 50 (49-55) | 50 (45-50) | 52 (42-55) | 45 (40-50) | 62 (50-70) | <0.001 |
| *Outcomes* |  |  |  |  |  |  |
| Failures, n (%) | 1 (1.9) | 1 (2.4) | 1 (12.5) | 1 (1.4) | 4 (25) | <0.001 |
| Early decannulation | 9 (17.3) | 19 (46.3) | 1 (12.5) | 15 (21.1) | 1 (6.3) | 0.003 |
| Average decannulation | 27 (51.9) | 15 (36.6) | 3 (37.5) | 31 (43.7) | 6 (37.5) | 0.618 |
| Late decannulation | 16 (30.8) | 7 (17.1) | 4 (50) | 25 (35.2) | 9 (56.3) | 0.040 |
| Lenght of stay, days | 74 (62-92) | 45 (32-63) | 95 (56-136) | 66 (52-94) | 79 (51-94) | 0.031 |
|  |  |  |  |  |  |  |

**Supplementary Table 9.** Clinical characteristics and outcomes of interest by participating center. Data are shown as frequencies (percentage) or median (inter-quartile range). COPD = Chronic Obstructive Pulmonary Disease; QsQ = Quantitative semi Quantitative score.


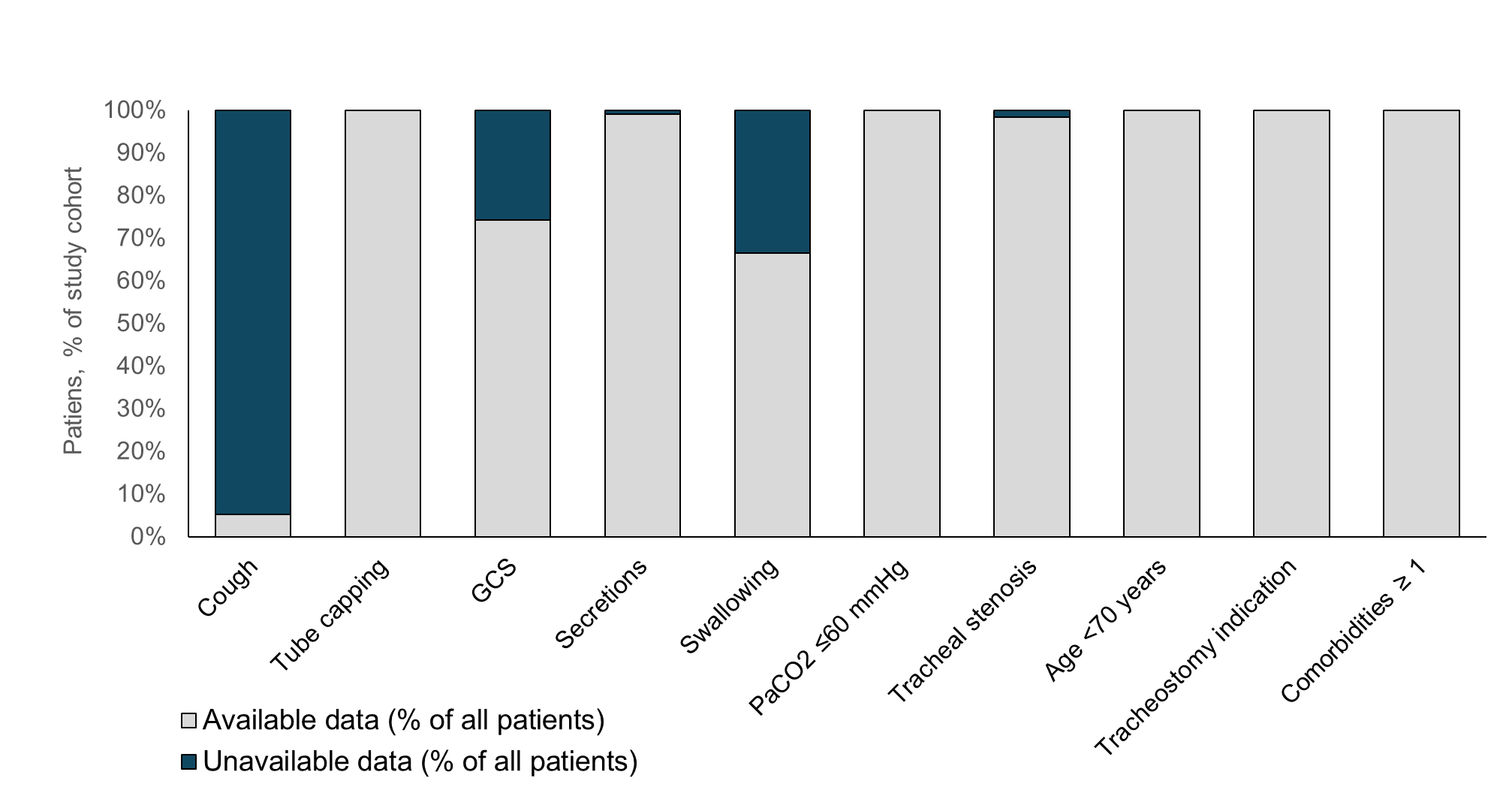


**Supplementary Figure 1.** Availability of variables in the study cohort used to assess major and minor QsQ criteria. Unavailable (blue) and available (light grey) histograms represent proportion of patients with or without the variable of interest among the whole study cohort (n = 191)


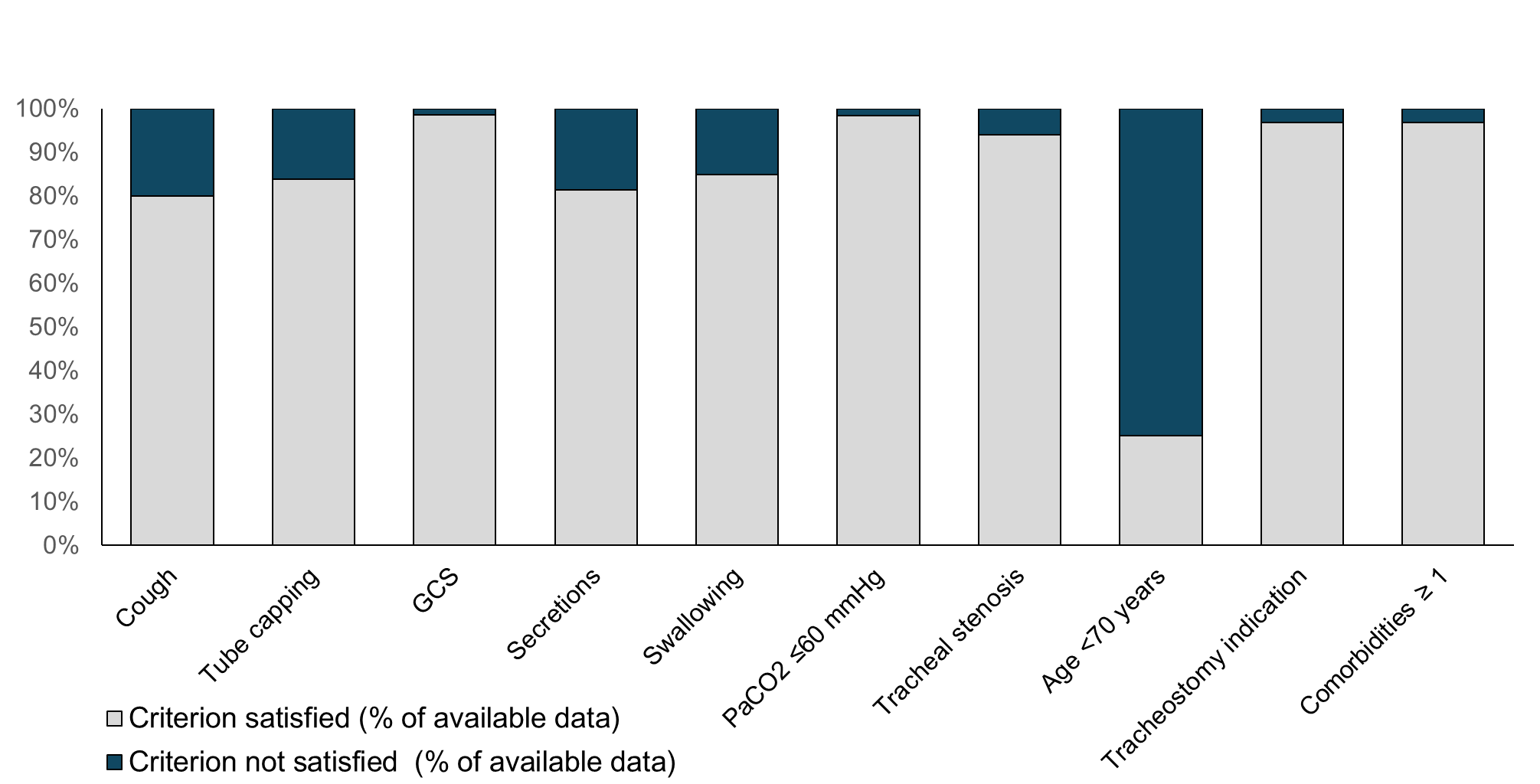


**Supplementary Figure 2**. Proportion of patients satisfying (light grey) major and minor QsQ criteria within the study cohort (n = 191) presented as prevalence of available data. Major QsQ criteria are cough validity and tube capping, while minor criteria are: level of consciousness, secretions control, preserved swallowing, a PaCO2 ≤ 60 mmHg, the absence of tracheal stenosis, an age <70 years old, a non surgical indication for tracheostomy and the absence of comorbidities. Please note that for cough assessment, the criterion (peak cough flow) was available in only 10 patients (5.2% of study cohort).
